# Post-Colonoscopy Perforation in a Patient With Ulcerative Colitis

**DOI:** 10.7759/cureus.83481

**Published:** 2025-05-04

**Authors:** Yash Shrivastava, Anand Krishnanand

**Affiliations:** 1 General Surgery, LN Medical College and Research Center, Bhopal, IND

**Keywords:** colonoscopy complications, colon perforation in inflammatory bowel disease, iatrogenic colon perforation, inflammatory bowel disease - ulcerative colitis, perforation of colon, perforation repair, pneumoperitoneum, post-colonoscopy colon perforation, primary repair of colon perforation

## Abstract

Ulcerative colitis, a chronic inflammatory bowel disease, renders the colonic mucosa particularly vulnerable to complications during endoscopic procedures. This case illustrates a serious complication following colonoscopy in active disease. A 25-year-old female presented with two weeks of hematochezia. Colonoscopy revealed severe active inflammation with ulcerations, friability, and spontaneous bleeding throughout the examined colon, consistent with chronic ulcerative colitis. Histopathology confirmed chronic architectural distortion with acute inflammatory infiltrates. Four hours post-procedure, the patient developed acute abdominal pain with guarding. Imaging demonstrated pneumoperitoneum with free subdiaphragmatic air. Emergency laparotomy identified a 4- to 5-mm perforation at the splenic flexure, correlating with an area of severe endoscopic inflammation. The site showed marked wall thinning without evidence of malignancy. Surgical management included primary closure with omental patch reinforcement and peritoneal lavage. This case demonstrates several key clinical considerations. First, it highlights the increased perforation risk during colonoscopy in active ulcerative colitis, particularly in severely inflamed segments. The splenic flexure's anatomical vulnerability may further predispose to perforation. Second, it emphasizes the importance of early recognition, where prompt surgical intervention likely contributed to the patient's successful recovery despite significant peritoneal contamination. Third, it illustrates the decision-making process for primary repair versus resection in iatrogenic perforations. The patient's postoperative course included appropriate antibiotic therapy and gradual nutritional advancement. At the time of discharge, she showed marked clinical improvement with resolving inflammatory markers. This case underscores the need for careful risk-benefit assessment when performing colonoscopy in active colitis and reinforces the value of preparedness for potential complications.

## Introduction

Colonoscopy has revolutionized the management of inflammatory bowel disease (IBD) since its introduction in the 1970s, serving as both a diagnostic and therapeutic cornerstone [1,2]. While generally safe, the procedure carries inherent risks, with perforation representing one of the most feared complications, particularly in patients with ulcerative colitis (UC), where mucosal friability and chronic inflammation amplify vulnerability [3,4]. This case report examines post-colonoscopy perforation (PCP) in a young UC patient, highlighting the interplay of procedural factors, disease severity, and immunosuppression that predispose to this life-threatening event. The overall incidence of colonoscopic perforation remains low (0.1-0.3% for diagnostic procedures and up to 3% for therapeutic interventions) [5,6], but IBD patients face disproportionately higher risks due to transmural inflammation, strictures, and corticosteroid use [7,8]. Mechanistically, perforations arise from (1) direct trauma from the endoscope or biopsy forceps, (2) barotrauma from excessive insufflation, or (3) therapeutic interventions such as stricture dilation or polypectomy [9,10]. In UC, the risk is further modulated by disease extent and activity, with severe pancolitis conferring the highest hazard [11]. Current guidelines emphasize caution when performing colonoscopy in active IBD, advocating for minimal air insufflation and avoidance of unnecessary biopsies in inflamed segments [12]. However, the procedure remains indispensable for assessing disease activity, detecting dysplasia, and managing complications such as strictures [13,14]. This paradox underscores the need for meticulous risk stratification - a challenge compounded by the lack of validated predictive tools. Emerging data suggest that concurrent anti-TNF therapy may mitigate perforation risk by reducing inflammation, whereas corticosteroids impair tissue healing and increase susceptibility [15,16]. The Montreal Classification categorizes UC by extent (E1-E3) and severity (S0-S3), with severe (S3) disease carrying a five-fold higher perforation risk during colonoscopy compared to quiescent cases [17,18]. Additionally, post-procedural perforations often manifest atypically in IBD patients, with delayed presentations (6-24 hours) due to masked symptoms from immunosuppression [19]. Such nuances demand heightened vigilance and prompt imaging (e.g., upright abdominal radiography or CT) when perforation is suspected [20]. This report details a case of PCP in a 25-year-old female with steroid-refractory UC, illustrating critical lessons in prevention, diagnosis, and management. By contextualizing our findings within the broader literature, we aim to refine safety protocols for high-risk IBD populations undergoing colonoscopy.

## Case presentation

**Figure 1:**
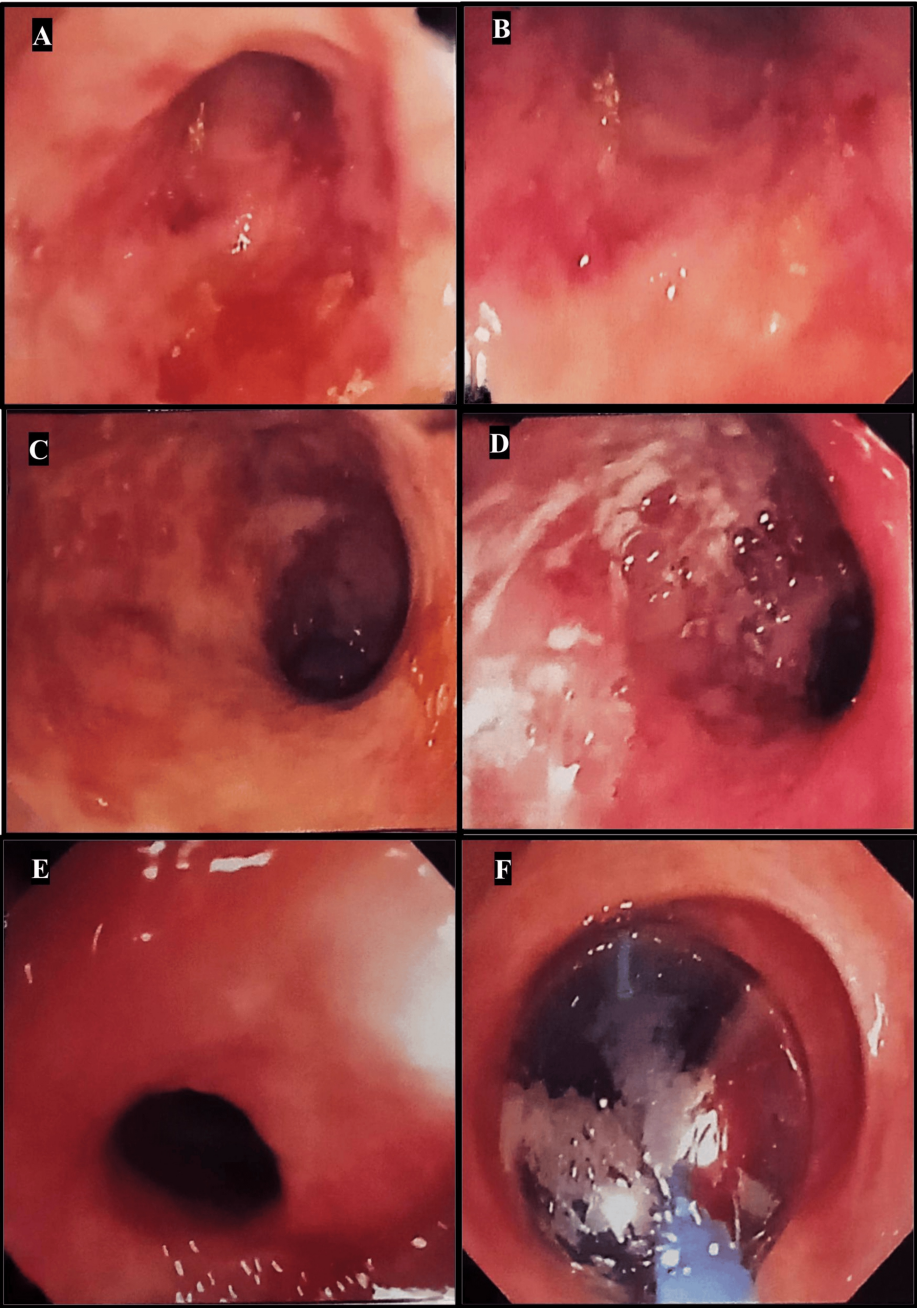
Therapeutic colonoscopy performed three months prior to presentation revealed proctosigmoiditis (A, B), IBD (C, D), and colonic stricture 30 cm from the anal verge, for which CRE balloon dilatation was performed (E, F). IBD, inflammatory bowel disease; CRE, controlled radial expansion

A 25-year-old woman was admitted to the Gastroenterology Department for watery diarrhea mixed with blood and mucous for 15 days. Her past history was significant for IBD for the past one year. Colonoscopy performed three months prior to presentation was suggestive of IBD with distal colonic stricture (Figure 1). Biopsy from the stricture was inconclusive.

**Figure 2:**
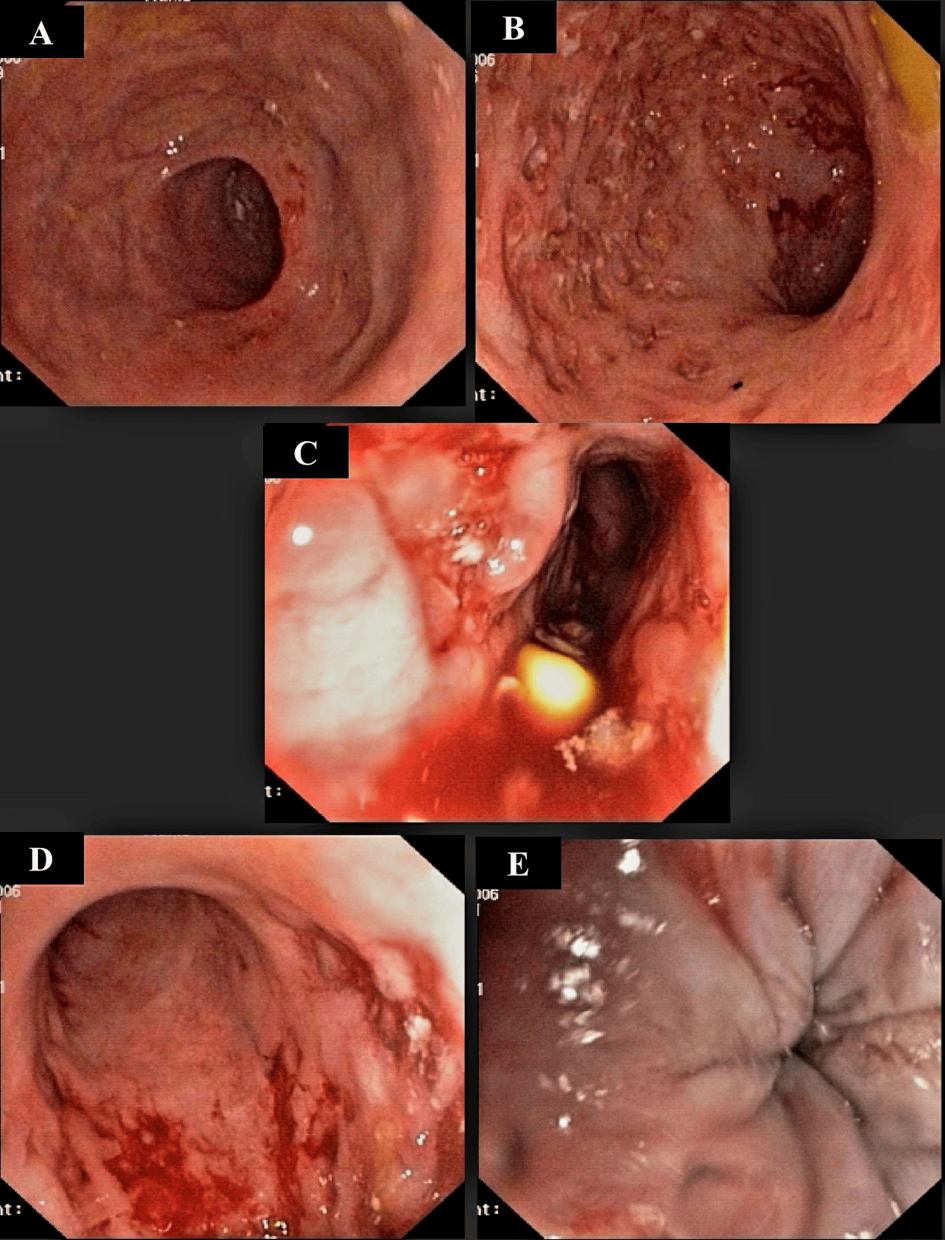
Colonoscopy findings on day 5 of admission were as follows: “Scope passed upto splenic flexure. Presence of multiple deep ulcers with mucosal friability and granularity along with loss of vascular pattern seen at the rectum, sigmoid colon, and descending colon (A-C). Biopsy taken from margins of ulcer at sigmoid colon for HPE. Rest of the mucosal study upto splenic flexure is normal (E). Small internal hemorrhoids seen (D). Impression - left sided active colitis? IBD-UC (biopsy taken)”. HPE, histopathological examination; IBD-UC, inflammatory bowel disease-ulcerative colitis

The patient was started on mesalazine and prednisolone. Repeat diagnostic colonoscopy on day 5 of admission was suggestive of active UC (Figure 2).

**Figure 3:**
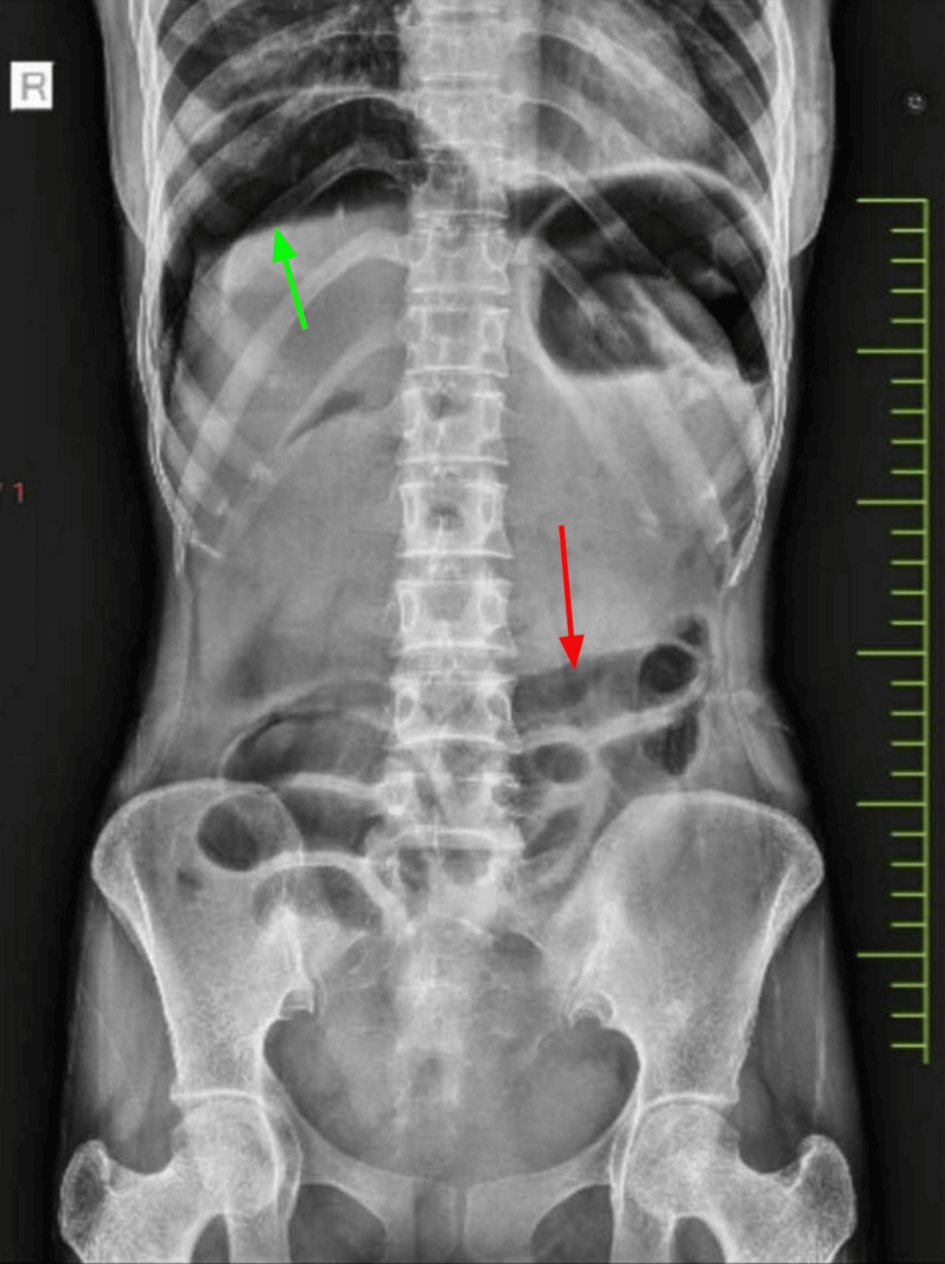
Abdominal radiograph performed 6 hours after colonoscopy revealed free gas under the right dome of the diaphragm (green arrow) with distended bowel loops (red arrow).

Gastroenterologist indicated that the colonoscopic procedure was uneventful and was performed safely. The patient was stable in the medicine ward till 4 hours after the procedure. She had two episodes of vomiting (gastric content) followed by generalized abdominal pain. She was shifted to the ICU in view of fever, tachycardia, and hypotension with a distended and tender abdomen. Abdominal radiograph was suggestive of bowel perforation (Figure 3).

The patient was reviewed by the general surgery unit on-call, and emergency laparotomy had to be planned in view of persistent features of perforation peritonitis. Significant laboratory findings were as follows: leukocytes 13,400/mm^3^, hemoglobin 9 g/dL, hematocrit 23.74%, and serum potassium 2.8 mmol/L (Table 1). This further confirmed the clinical findings.

**Table 1:** Lab Investigations suggestive of septicemia in view of evolving perforation peritonitis.

Laboratory Parameter	Patient Value	Reference Range
Total leukocyte count	13,400/mm^3^	4,000-11,000/mm^3^
Hemoglobin	9 g/dL	11.5-15.5 g/dL (female)
Hematocrit	23.74%	36-48% (female)
Serum potassium	2.8 mEq/L	3.5-5 mEq/L

The patient was kept nil orally. A nasogastric tube was inserted for gastric decompression. Intravenous (IV) fluids, IV broad-spectrum antibiotics, IV spasmolytics, and IV proton pump inhibitors were started, and electrolyte correction was done.

**Figure 4:**
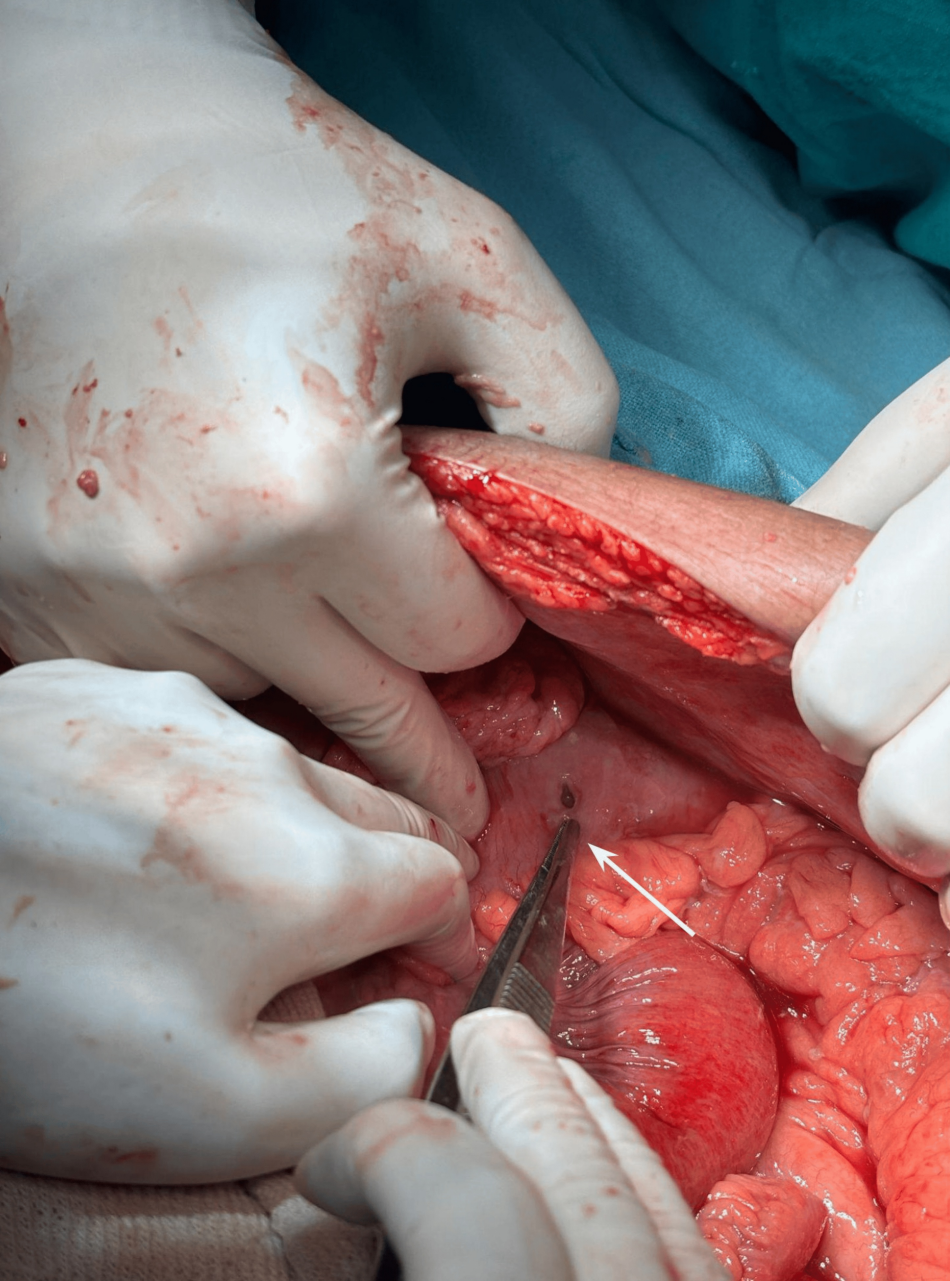
Exploratory laparotomy findings showed perforation in the splenic flexure of the colon marked by the tip of forceps (white arrow).

**Figure 5:**
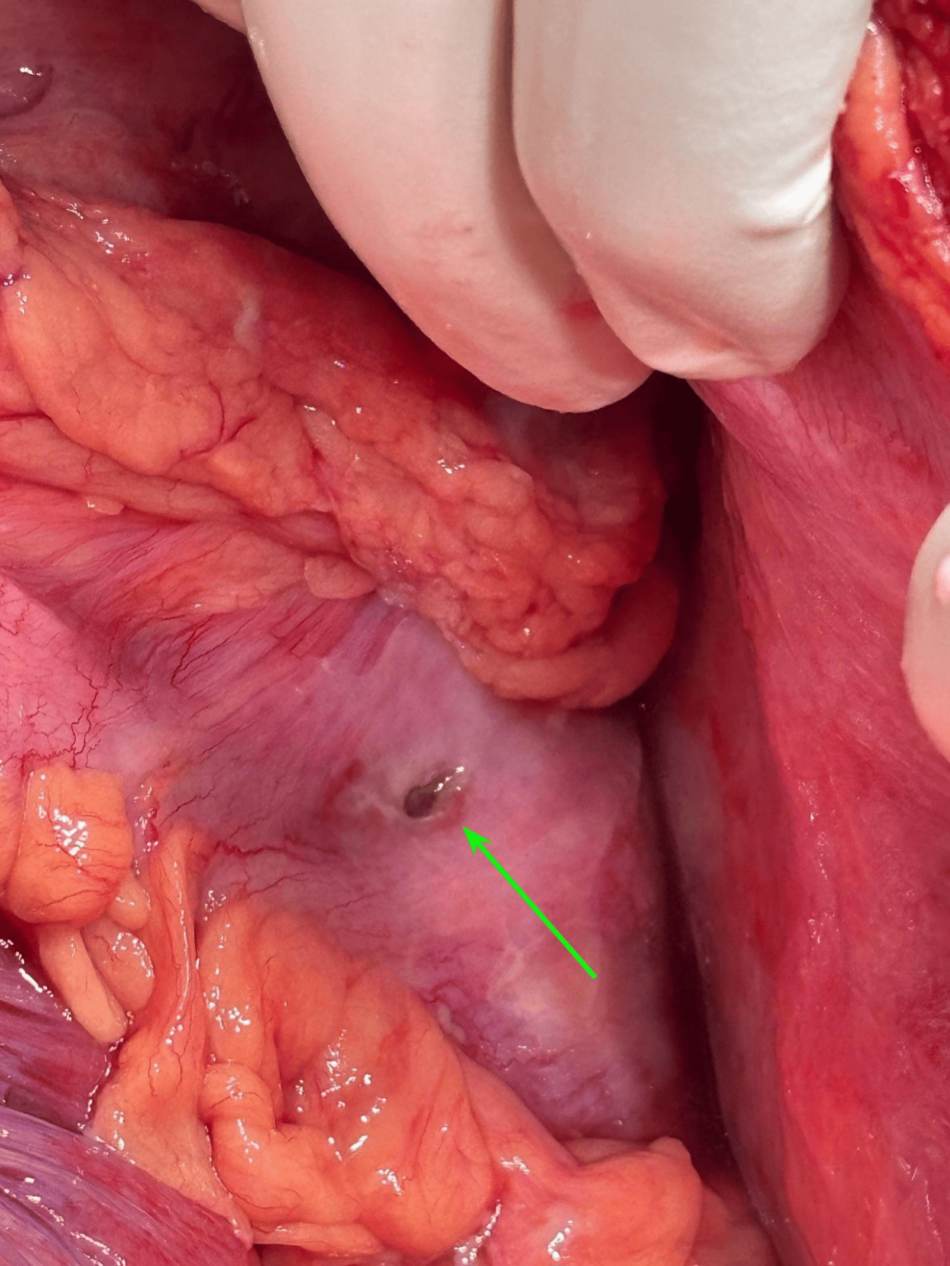
After clearance of purulent contents, the transmural perforation was localized on the antimesenteric border of the splenic flexure of the colon, with extravasation of feculent discharge noticed on the margins (green arrow).

With informed consent and necessary preparations, exploratory laparotomy with a longitudinal midline incision was performed on day 6 of admission. Intraoperative findings were as follows: there was purulent peritoneal fluid approximately 3 liters in volume, multiple pus flakes over the bowel surface, and solitary transmural perforation of 5 mm diameter along the antimesenteric border of the splenic flexure of colon with feculent discharge (Figures 4, 5).

We performed a double-layered closure, commencing with a continuous 3-0 Vicryl suture. Subsequently, an external layer of interrupted seromuscular 2-0 Vicryl sutures followed by an omental patch was applied. A thorough bowel wash with warm saline was given, and a pelvic drain was kept. Tissue from margin of the perforation was sent for HPE. The patient was kept nil by mouth and treated with IV fluids and antibiotics. Except for slight abdominal pain during the first 24 hours, there were no signs of peritonitis or abdominal distension. The initial white blood cell count was 13,200/mm^3^ (normal range: 4,600-10,500/mm^3^), which declined to 10,200/mm^3^ after 48 hours. Abdominal pain resolved by day 2, and her oral feeding was resumed by day 3. Drain removed on day 5. She was transferred to the Gastroenterology side for further treatment of UC.

## Discussion

This case of PCP in a 25-year-old female with severe UC underscores critical challenges in endoscopic management of IBD. Our patient’s transmural perforation at the splenic flexure - a site vulnerable to barotrauma due to acute angulation - exemplifies the multifactorial etiology of PCP, where procedural mechanics (e.g., air insufflation pressure) and disease-specific factors (e.g., mucosal friability) converge [5,6,9]. Below, we contextualize these findings within three key domains: risk stratification, management paradigms, and preventive strategies.

Risk stratification in IBD patients

The incidence of PCP in UC patients (0.5-1.5%) is markedly higher than in non-IBD populations (0.1-0.3%) [7,8]. Our case aligns with studies identifying severe endoscopic activity (Mayo score 3), extensive colitis (Montreal E3), and concurrent corticosteroid use as independent risk multipliers [11,18]. Notably, the patient’s prior stricture dilation - a known hazard [10,20] - may have further compromised bowel wall integrity. These observations corroborate Silverberg et al.’s findings that steroid-refractory UC patients exhibit a 4.2-fold increased perforation risk compared to those on biologics [16], likely due to impaired tissue repair and masked symptoms of peritonitis [19]. Genetic predisposition may also play a role, though this remains underexplored. The Montreal Classification’s phenotypic framework (e.g., structuring vs. penetrating disease) [17] could aid pre-procedural risk assessment, particularly in patients with long-standing UC (>10 years) who develop fibrosis-related complications [15].

Management dilemmas

Our patient’s emergent laparotomy with primary repair reflects the gold standard for overt perforations with peritonitis [6,12]. However, controversies persist regarding conservative management for microperforations (e.g., post-polypectomy coagulation syndrome) or localized leaks without sepsis [9,12]. Terheggen et al. reported successful non-operative management in 68% of IBD-related perforations [13], but this approach demands (a) rapid diagnosis (upright abdominal radiography, as utilized here, detects pneumoperitoneum in 85% of cases, while CT offers superior sensitivity for retroperitoneal leaks) [5,20] and strict monitoring (leukocytosis [>12,000/mm³] and persistent tachycardia [>100 bpm] should trigger surgical consultation [6,19]. Notably, our patient’s delayed presentation (4 hours post-procedure) mirrors Rubin et al.’s cohort, where immunosuppression masked symptoms in 41% of cases [9]. This underscores the need for extended post-colonoscopy observation in high-risk IBD patients.

Preventive strategies

To mitigate PCP risk, we propose the following protocol, synthesized from guideline recommendations [3,12] and outcome studies [7,16].

Pre-procedural Optimization

Defer elective colonoscopy in active steroid-dependent UC (Mayo score ≥2) until biologic induction achieves remission [16]. Utilize CO₂ insufflation (rather than air) to reduce barotrauma risk, as demonstrated by Williams et al. [6].

Technical Modifications

Employ pediatric colonoscopes in severe colitis to minimize mechanical stress [13]. Avoid biopsies in deeply ulcerated areas unless absolutely necessary [12].

High-Risk Scenario Planning

For strictures, prefer balloon dilation over electrocautery, with a maximum diameter ≤15 mm to avoid transmural injury [10,20]. Schedule therapeutic procedures at tertiary centers with surgical backup [8].

Limitations and future directions

While our case reinforces established risk factors, generalizability is constrained by its single-center nature. Larger, multicenter registries, such as those analyzed by Arora et al. [4], are needed to validate predictive models incorporating genetic markers (e.g., NOD2 variants) [17] and assess the impact of newer biologics (e.g., ustekinumab) on perforation risk [16]. Additionally, artificial intelligence tools for real-time perforation risk scoring during colonoscopy warrant exploration, particularly in IBD populations [14]. This case highlights PCP as a potentially catastrophic complication of colonoscopy in UC, where disease severity and iatrogenic factors interact synergistically. A triad of risk awareness, technical adaptation, and individualized management is essential to improve outcomes. Future research should focus on risk-prediction algorithms and safer therapeutic techniques for this vulnerable population.

## Conclusions

PCP represents a significant concern in patients with inflammatory bowel conditions, particularly those with active mucosal disease. The clinical scenario presented illustrates how underlying intestinal inflammation can predispose to procedural complications, emphasizing the need for heightened awareness during both pre-procedural planning and post-procedural monitoring. When such complications occur, timely recognition and appropriate intervention become paramount, with surgical management often playing a crucial role in cases showing systemic involvement. Clinical decision-making must carefully consider individual patient factors including disease activity and medication regimens, which may influence both risk and management approaches. This situation underscores the value of meticulous endoscopic technique and comprehensive patient assessment prior to undertaking diagnostic procedures. Maintaining vigilance for potential complications and ensuring rapid access to necessary interventions can significantly impact clinical outcomes. For specialists managing chronic intestinal inflammation, these considerations become particularly relevant when determining the timing and approach to necessary diagnostic evaluations. The experience described reinforces the importance of tailored patient care and coordinated management when addressing complex clinical situations. Ongoing advancements in procedural techniques and peri-procedural care continue to refine our ability to safely evaluate patients with compromised intestinal integrity, though the fundamental need for careful risk-benefit assessment remains constant. This clinical example serves to highlight the delicate considerations involved when performing invasive diagnostic procedures on patients with vulnerable mucosal surfaces, where the imperative for accurate diagnosis must be carefully balanced against procedural risks.
